# Development and technical evaluation of a reusable 3D-printed cytospin funnel for enlarged cytospin generation

**DOI:** 10.1186/s13104-026-08021-z

**Published:** 2026-09-29

**Authors:** Christopher Herz, Nicole Weiß, Carlo Mümmler, Michael Gerckens, Nikolaus Kneidinger, Jürgen Behr, Nikolaus A. Haas, Marina Nagel

**Affiliations:** 1 Department of Cardiac Surgery, LMU University Hospital, LMU Munich, Munich, Germany; 2 Chair of Medical Materials and Implants, Technical University Munich, Munich, Germany; 3 DZHK (German Centre for Cardiovascular Research), partner site Munich Heart Alliance, Munich, Germany; 4 Department of Medicine V, Comprehensive Pneumology Center (CPC), LMU University Hospital, LMU Munich, Member of the German Center of Lung Research (DZL), Munich, Germany; 5 Institute of Lung Health and Immunity (LHI), Comprehensive Pneumology Center (CPC), Helmholtz Munich, Member of the German Center of Lung Research (DZL), Munich, Germany; 6 Division of Respiratory Medicine, Department of Internal Medicine, Lung Research Cluster, Medical University of Graz, Graz, Austria; 7 Department of Pediatric Cardiology and Intensive Care, LMU University Hospital, Munich, Germany

**Keywords:** Cytology, Cytospin, Funnel, 3D printing, Effusion cytology

## Abstract

**Objective:**

Cell fixation on a microscope glass slide is a standard technique for evaluating cellular morphology in a wide range of biosamples. Cytofunnels are used in a cytocentrifuge to direct cells onto the glass slide and generate a “cytospin”. Commercial cytofunnels have a fixed design with limited cytospin areas, being mostly single-use and are costly. The goal of this study was to design and manufacture a 3D-printed, customized cytofunnel that is cost-efficient, reusable and generates a larger cytospin area. We used an iterative design process using computer-aided design (CAD) software and stereolithography (SLA) printing with biocompatible resin. The funnel prototypes were subjected to a technical evaluation using two independent human pleural effusion specimens, as well as through repeated exposure to two commonly used disinfectants.

**Results:**

The 3D-printed cytospin funnel featured larger outlet diameters, increasing the cytospin area approximately threefold with a total manufacturing time of 24 h. The presented reusable funnel design enabled enlarged cytospin areas and increased practical cell-loading capacities under the tested conditions and may support future digital cytology and high-throughput analysis workflows. The published STL file may provide an accessible, sustainable alternative for laboratories, especially in resource-limited settings and may serve as a basis for further customization.

**Supplementary Information:**

The online version contains supplementary material available at https://doi.org/10.1186/s13104-026-08021-z.

## Introduction

One of the standard procedures of cytopathological examination is the evaluation of the cellular content of (effusion) fluids on a microscopic glass slide, a cytospin [1]. To create these cytospins, the examination material is fixed to a glass slide by centrifugal force using a cytocentrifuge and a cytofunnel [2]. Commercial cytofunnels are typically single-use, thus generating high costs and plastic waste. Further, due to the limited outlet diameter only a small fraction of the material can be used. In previous decades this was not regarded as a problem as human cytopathologist resources were the limiting factor for subsequent cytospin analysis. Recent advances in digital pathology and computer vision-based image analysis increasingly enable automated assessment of large numbers of cells on cytological slides [3]. Larger cytospin areas and increased cell loading capacities may therefore support downstream cytological evaluation and digital analysis workflows [4, 5].

Additive manufacturing using stereolithography (SLA) has significantly advanced in recent years [6] and enabled applications such as 3D-printed dental and vascular prostheses. This study aimed to develop and technically assess a reusable, customized 3D-printed cytospin funnel compatible with conventional cytocentrifuge systems and capable of generating larger cytospin areas.

## Materials and methods

The key design requirements for the custom-made cytospin funnel were compatibility with standard cytocentrifuges, enlarged cytospin surface areas, higher loading capacities, and reusability after chemical disinfection.

**Fig. 1:**
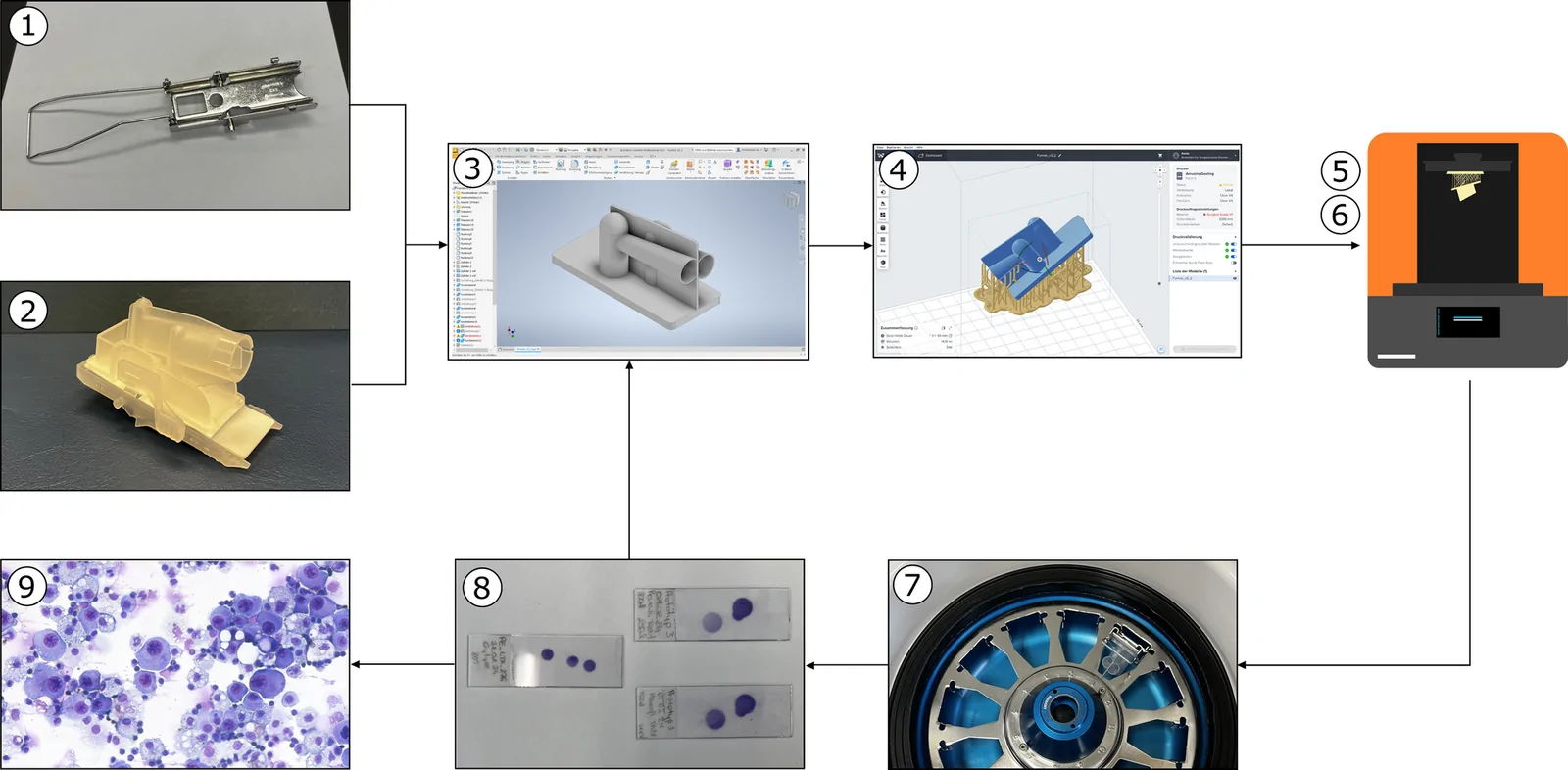
Iterative design, manufacturing steps, and testing of the novel cytospin funnel prototype: **1** Standardized centrifuge metal clamps, **2** commercially available double funnel serving as a template for the adapted design, **3** prototype design in CAD software and subsequent export as STL file, **4** preparation of the prototype model for 3D printing using a slicing software, **5** additive manufacturing process using a SLA printer with a biomedical photopolymer, **6** post-processing of the 3D printed part including removal of support structures, washing, and curing, **7** disinfection and integration of the prototype funnel into the centrifuge to fix the sample material of interest onto the sample slide, **8** macroscopic evaluation of the cell monolayer, **9** validation and microscopic evaluation of the cell monolayer achieved by cytocentrifugation technique using the funnel prototype

The development of the funnel followed an iterative prototyping workflow involving repeated design adaptation and functional testing (Fig. 1). Maximum base size and height were confined by the standardized metal clamps (Edelstahlclip für Zelltrichter, Tharmac^®^ GmbH) of the centrifuge (Thermo Shandon Cytospin 4, Thermo Fisher Scientific). Prototype funnels were designed using computer-aided design (CAD) software (Inventor Professional Pro 2023, Autodesk Inc.). To manufacture the models, a commercial SLA printer (Form 3B, Formlabs Inc.) was used with a rigid, biomedical resin (Surgical Guide v1, Formlabs Inc.). Post-processing included removal of the support structures and washing of the parts in 99,0% Iso-2-propanol for 20 min. The parts were dried at room temperature for 12 h. Subsequently, the models were cured for 30 min at 60 °C using a commercial curing chamber (Form Cure, Formlabs Inc.).

The prototype funnels were evaluated under representative laboratory conditions using two independent human pleural effusion specimens. Each prototype was evaluated in three to five cytospin procedures, with different cell concentrations ranging from 20,000 to 800,000 cells used for each one. The commercial and 3D-printed funnels were evaluated using identical sample preparation and cytocentrifugation conditions (550 rpm for 10 min) in the same cytocentrifuge, followed by identical downstream slide processing. The sole parameter that exhibited variation between the two funnel designs was the volume of cell suspension loaded into the funnel, thereby reflecting their divergent loading capacities. The technical objective was to generate via cytospin cell monolayers with adequate distribution and minimal cell overlap. The cytospin areas were calculated from the circular outlet dimensions using the standard formula for the area of a circle (A = πr²). During the iterative technical evaluation, the practical cell-loading capacities were estimated by testing increasing cell numbers and determining the highest cell numbers that still resulted in adequately distributed cell monolayers without excessive cell overlap or aggregation. Reusability was technically assessed by repeated exposure of the funnels to two commonly used types of disinfectants (Korsolex^®^ Extra, Bode Chemie GmbH, Hamburg, Germany; Sekusept™ aktiv, Ecolab Deutschland GmbH, Monheim am Rhein, Germany) followed by visual inspection for macroscopic deformation or functional impairment. Due to the exploratory and technical nature of the project, as well as the focus on the proof-of-concept, no formal statistical analysis was conducted.

## Results

Three generations of prototypes were designed and additively manufactured in an iterative design adaptation process. The final prototype included two funnels with slight offset of the outlets (Supplementary Fig. 1). The maximum filling volumes of the funnels were 450 µl of the cell suspension compared to 250 µl for the conventional double funnel. Outlet diameters of the 3D-printed funnel were 9 mm compared to 5 mm of the conventional double funnel. Iterative design resulted in precise fit within the standard metal clamps, ensuring sufficient pressure on the cytospin funnel and transfer of cell material to the slides. Representative cytospin experiments demonstrated monolayer areas of approximately 64 mm² compared to approximately 20 mm² per outlet achieved with conventional double funnels (Supplementary Fig. 2). The estimated practical cell-loading capacity under the tested conditions ranged from approximately 400,000 to 800,000 cells for the 3D-printed funnel, compared with approximately 100,000 to 200,000 cells for the conventional cytospin funnel. This was achieved while maintaining an adequately distributed cell monolayer without excessive cell overlap or aggregation. The manufacturing costs for a reusable 3D-printed cytospin funnel were approximately 4.7 USD, in comparison to 2.8–4.1 USD for commercially available single-use double funnels [7]. It was established that exposure to the tested disinfectants and liquid media did not result in any observable macroscopic deformation or relevant functional impairment of the funnels during routine use. The total production time for a single prototype funnel was approximately 24 h, including approximately 3.5 h of active SLA printing time.

## Discussion

Cytocentrifugation is a standard technique to transfer cells from cell suspensions in biological samples onto a microscopic slide for subsequent analysis [1]. Commercially available funnels often have a limited loading volume and are designed for single use. This contributes to high consumable costs and restricted cytospin areas. In this technical proof-of-concept study, we developed and evaluated a customized, reusable 3D-printed cytospin funnel compatible with standard cytocentrifuge systems. The presented design enabled substantially larger cytospin areas compared to conventional double-funnel systems while maintaining compatibility with routine laboratory workflows.

The proposed iterative workflow enabled the manufacturing of several funnel prototypes and implementation of design adaptations. The initial pure upscaling of a standard double funnel serving as template led to large funnel openings and thus to large losses of liquid during centrifugation. Outlet diameters and model wall thickness were adjusted as the first prototype could not be used with standard metal clamps due to insufficient contact pressure.

The proposed design of the final prototype’s funnel openings and increased outlet diameters allowed for the incorporation of larger filling volumes and the generation of a larger cytospin size, which could be advantageous for the detection of rare target cells, such as malignant cells in low-cellularity specimens. Emerging digital pathology and computer vision approaches may further benefit from larger cytospin areas and increased cellular distribution, potentially facilitating automated high-throughput analysis workflows.

We believe that the STL file published with this article and suited for 3D printing might allow a greater cost-effectiveness and sustainability in laboratories and clinics, particularly in resource-limited settings, and could potentially serve as a basis for further adaptation to specific laboratory needs. The STL file is freely accessible via the institutional 3D laboratory website.

## Limitations

The present study is subject to several limitations. The results obtained are derived from exploratory technical tests and iterative prototyping, rather than from a formally controlled comparative study. The technical evaluation was constrained to two independent samples of human pleural effusions and three to five cytospin procedures per prototype, which restricts the generalisability of the results to other sample types and laboratory conditions. Moreover, a systematic quantitative assessment of diagnostic sensitivity, cell integrity and long-term material stability is yet to be conducted. Further studies with larger and more diverse sample sets are required to evaluate the performance of the 3D-printed funnel in routine cytological applications.

## Supplementary information

Below is the link to the electronic supplementary material.

Supplementary Material 1: Technical drawing of the final prototype.

Supplementary Material 2: Macroscopic and microscopic evaluation of cell monolayers‘ qualitative area and cell density achieved by cytocentrifugation using a commercially available funnel and the novel 3D-printed cytospin funnel: A, B) microscopic cell density and distribution achieved with a conventional funnel and a sample volume of 250 µl (estimated practical cell-loading capacity of 100,000 to 200,000 cells under the tested conditions) and a magnification of 10x and 20x; C, D) microscopic cell density and distribution achieved with the novel funnel prototype and a sample volume of 450 µl (estimated practical cell-loading capacity of 400,000 to 800,000 cells under the tested conditions) and a magnification of 10x and 20x; E) exemplary macroscopic cell monolayer areas achieved with the 3D-printed funnel (top row) and the commercial funnel (bottom row).

## Data Availability

The STL file supporting this study will be made freely available via the institutional 3D laboratory website upon publication of this manuscript. The download will be available at: https://www.lmu-klinikum.de/kinderkardiologie/forschung-und-lehre/forschung/unsere-arbeitsgruppen/ag-3d-druck/angebot/3d5d486e59c03789.

## Abbreviations

SLA: stereolithography
CAD: computer-aided design
