# Supplementary material for: Development and technical evaluation of a reusable 3D-printed cytospin funnel for enlarged cytospin generation
Source: BMC Res Notes. 2026 Sep 29;19:366. doi: 10.1186/s13104-026-08021-z (PMC13621705; doi:10.1186/s13104-026-08021-z)
Supplement: Supplementary file 1 [file 13104_2026_8021_MOESM1_ESM.pdf]

BAUTEILLISTE		
OBJEKT	ANZAHL	BAUTEILNUMMER
1	1	Funnel_v3_2

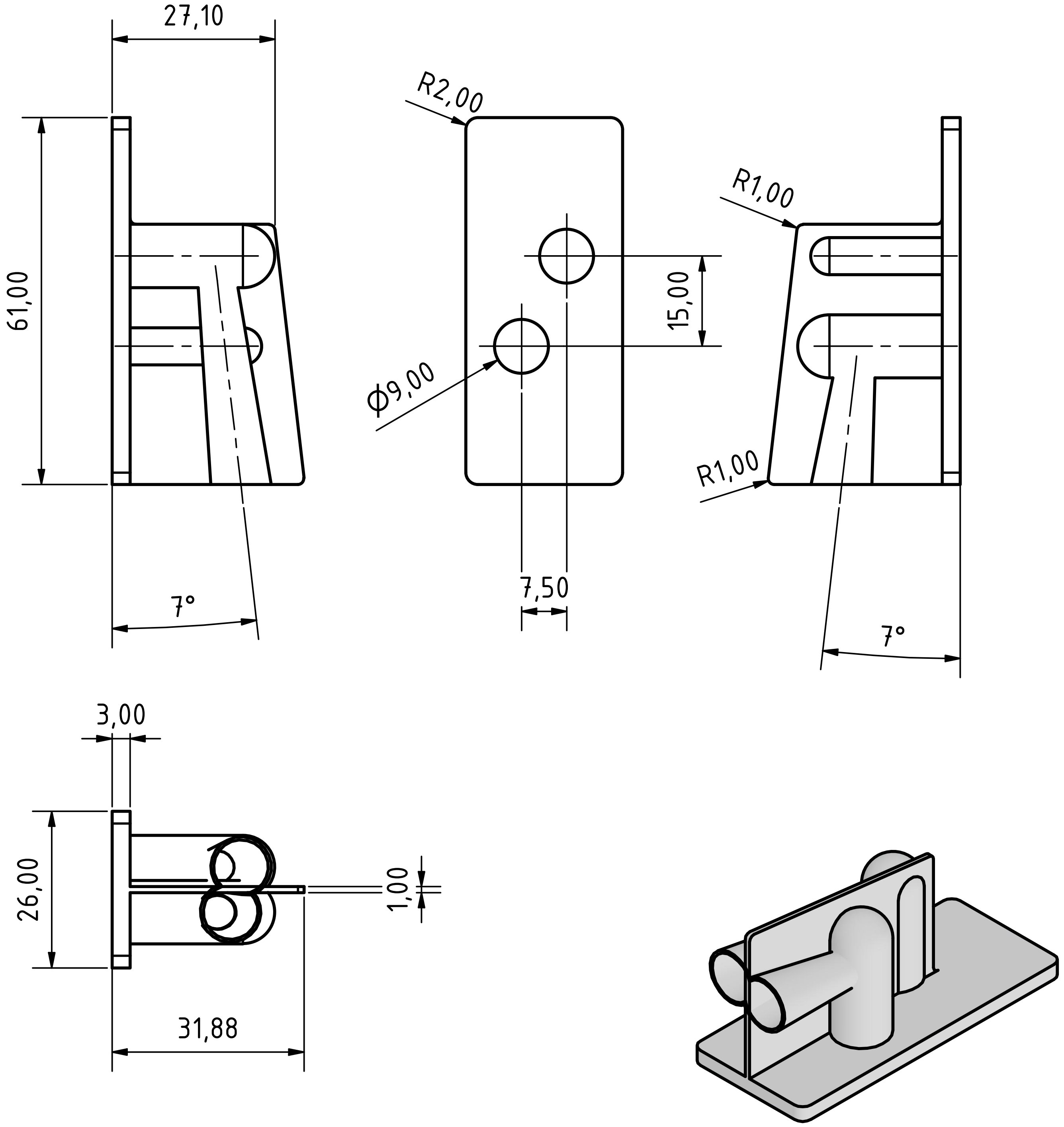

	Datum	Name
Gezeich	30.10.2024	chher
Kontroll		
Norm		

Status	Änderungen	Datum	Name

Funnel_v3_2

1

A4
