# Supplementary material for: Development and technical evaluation of a reusable 3D-printed cytospin funnel for enlarged cytospin generation
Source: BMC Res Notes. 2026 Sep 29;19:366. doi: 10.1186/s13104-026-08021-z (PMC13621705; doi:10.1186/s13104-026-08021-z)
Supplement: Supplementary file 2 [file 13104_2026_8021_MOESM2_ESM.pdf]

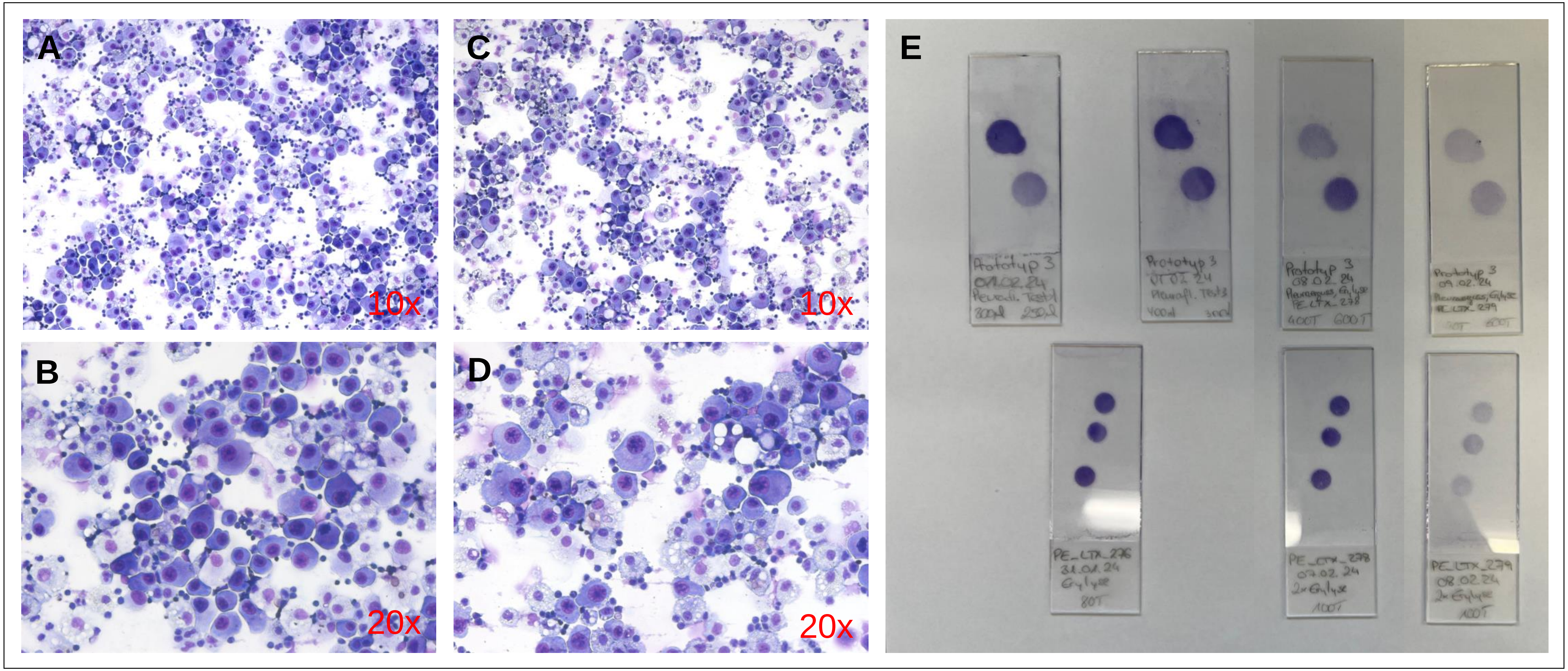

Supplementary Fig. 2: Macroscopic and microscopic evaluation of cell monolayers‘ qualitative area and cell density achieved by cytocentrifugation using a commercially available funnel and the novel 3D printed cytospin funnel: **A**, **B**) microscopic cell density and distribution achieved with a conventional funnel and a sample volume of 250 µl (maximum evaluable cell number of 100.000 to 200.000 cells) and a magnification of 10x and 20x, **C**, **D**) microscopic cell density and distribution achieved with the novel funnel prototype and a sample volume of 450 µl (maximum evaluable cell number of 400.000 to 800.000 cells) and a magnification of 10x and 20x, **E**) exemplary macroscopic cell monolayer areas achived with the 3D printed funnel (top row) and the commercial funnel (bottom row).
